# Incorporating obstetrical simulation into physician assistant didactic curriculum

**DOI:** 10.1186/s12909-025-08342-w

**Published:** 2025-12-29

**Authors:** Ashley D. Griffin, Nicole K. Daher, Jolene E. Bohensky

**Affiliations:** 1 Philadelphia College of Osteopathic Medicine, Department of Physician Assistant Studies, 625 Old Peachtree Rd. NW, Suwanee, GA 30024 USA; 2 Philadelphia College of Osteopathic Medicine, Department of Physician Assistant Studies, Rowland Hall, Suite 207 4190 City Avenue, Philadelphia, PA 19131 USA

**Keywords:** Obstetrical simulation, Labor and delivery, Vaginal delivery, Problem-based learning, High fidelity manikin, Clinical skills

## Abstract

**Background:**

Ensuring physician assistant (PA) students are adequately prepared and exposed to women’s health competencies is a requirement for PA programs regulated by Accreditation Review Commission on Education for Physician Assistants (ARC-PA). Over the years, there has been a growing shortage of clinical rotation sites in women’s health, particularly those offering obstetrical experiences. Medical simulation is being suggested as an alternative method to supplement a student’s experience and to bridge knowledge gaps. Program faculty at the Philadelphia College of Osteopathic Medicine Physician Assistant Program developed, implemented, and piloted a simulated vaginal delivery experience performed on high-fidelity manikins for students in the didactic year. The labor and delivery experience enhanced clinical skills for upcoming clinical rotations through case-based learning.

**Methods:**

During this pilot study, high-fidelity childbirth simulation manikins were utilized to offer didactic students a technologically innovative learning experience. Didactic students completed pre- and post-surveys evaluating their experience, knowledge, perspective and skill sets acquired in the labor and delivery setting. Conventional qualitative content analysis was applied to students’ optional narrative comments from the post-simulation survey.

**Results:**

The pre and post-survey response rate was 98% and 74% respectively. Survey results demonstrated a positive student experience. Survey conceptually matched categories yielded high response regarding reinforcing clinical knowledge (93.4% pre-survey, 94.3% post-survey), hands-on/practical skills (92.3% pre-survey, 90.0% post-survey), and understanding/applying skills (93.4% pre-survey and 92.8% post-survey). Conventional qualitative content analysis revealed two prominent themes of *Preparation for Clinical Rotations* and *Enhancing Knowledge Beyond the Classroom.*

**Conclusion:**

Incorporating obstetrical simulation into the PA curriculum improves student preparation and comfort prior to real-life situations during clinical rotations. Data supports the use of problem-based obstetrical simulation as a valuable teaching alternative. Future research should evaluate the potential improvements in standardized testing scores in the field of women’s health after completing this problem-based learning simulation activity.

**Supplementary Information:**

The online version contains supplementary material available at 10.1186/s12909-025-08342-w.

## Background

Physician assistant (PA) education has evolved dramatically since the inception of the first formal physician assistant training program, developed in 1965 at Duke University. Currently, the PA curriculum averages 27 months in length which includes didactic curriculum followed by more than 2,000 h of clinical rotations [1]. The Accreditation Review Commission on Education for Physician Assistant (ARC-PA) requires PA programs to provide a diverse range of clinical practice experiences that provide adequate exposure and competencies of patients seeking medical care across a patient’s life span, women’s health, conditions requiring surgical management, and care for behavioral and mental health conditions [2]. ARC-PA requires that clinical experiences include the following disciplines: family medicine, internal medicine, general surgery, pediatrics, obstetrics/gynecology (OB/GYN), and behavioral and mental health care [2]. 

Over the past several years, there has been a growing shortage of clinical rotation sites, particularly in the field of women’s health, obstetrics and gynecology. A recent Physician Assistant Education Association (PAEA) appropriations testimony states, “Nearly 75% of PA programs indicate that it is either harder or much harder to secure clinical rotations in obstetrics/gynecology than prior to the COVID-19 pandemic.”[3] Additionally, within the discipline of women’s health, finding clinical sites that offer physician assistant students experience in obstetrical care has become increasingly more challenging. Due to this shortage, medical simulation is being suggested as an alternative method to supplement a student’s educational experience, help bridge knowledge gaps, and improve maternal and infant health outcomes. Simulation-based training (SBT) is a highly effective educational tool utilized in medical-based training to improve performance and promote skill retention [4]. Obstetrical simulation has been proven to increase improve outcomes such as knowledge, interprofessional skills, leadership capabilities, and decision-making during obstetrical encounters[5].

Due to the growing difficulty with acquiring obstetrical clinical experiences for Philadelphia College of Osteopathic Medicine Physician Assistant students, program faculty developed a new pilot obstetrical intrapartum simulated vaginal delivery experience for didactic students to improve preparation and comfort prior to progressing to clinical rotations and entering the workforce with the goal of improving patient outcomes. The didactic students who participated in this pilot experience expressed through surveys an overwhelming positive experience. With the promising results shown with this activity, our goal is to inspire other physician assistant educators to consider a similar approach to help supplement instruction.

## Methods

**Fig. 1:**
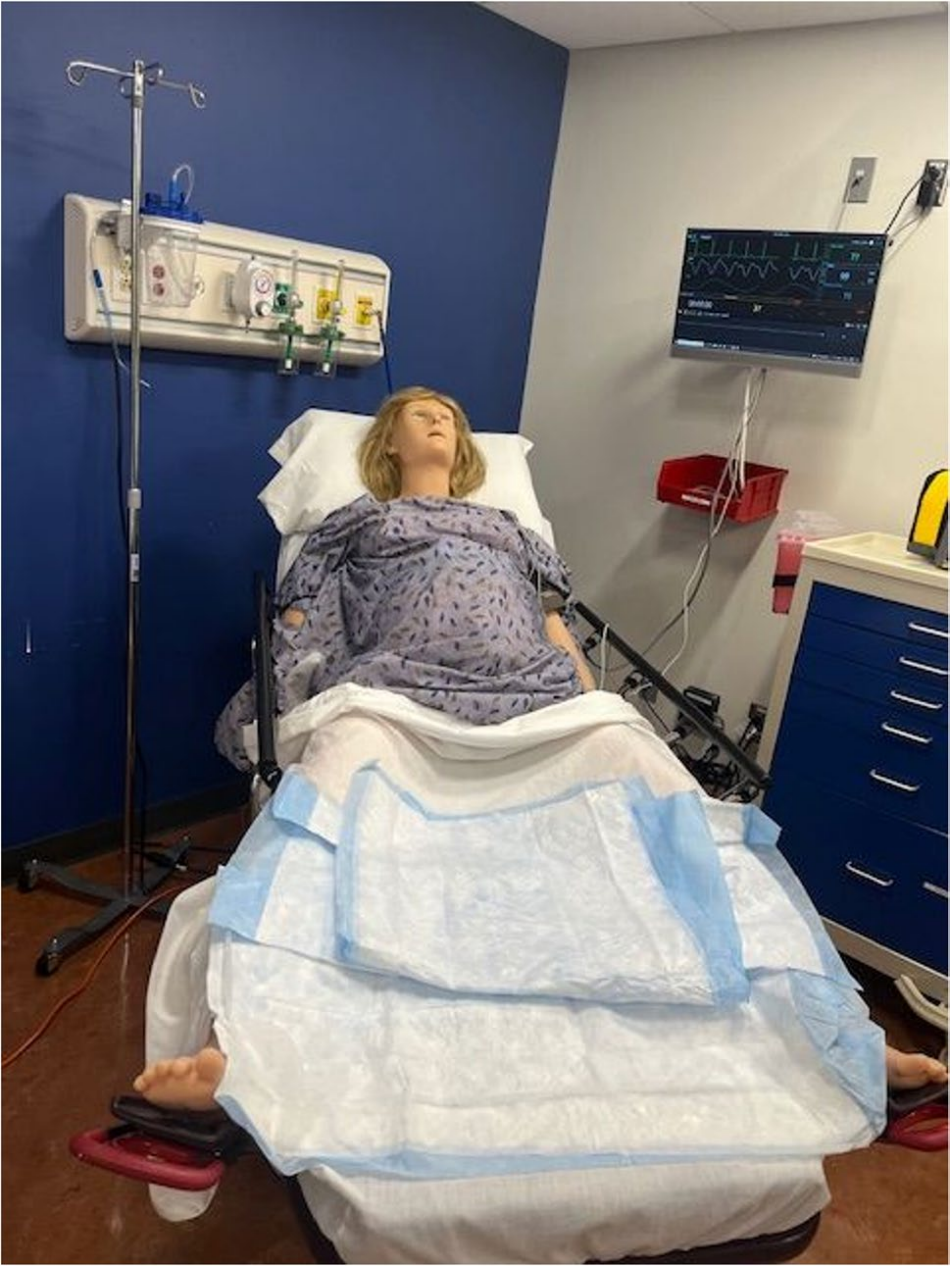
High fidelity model- VICTORIA S2200

**Fig. 2:**
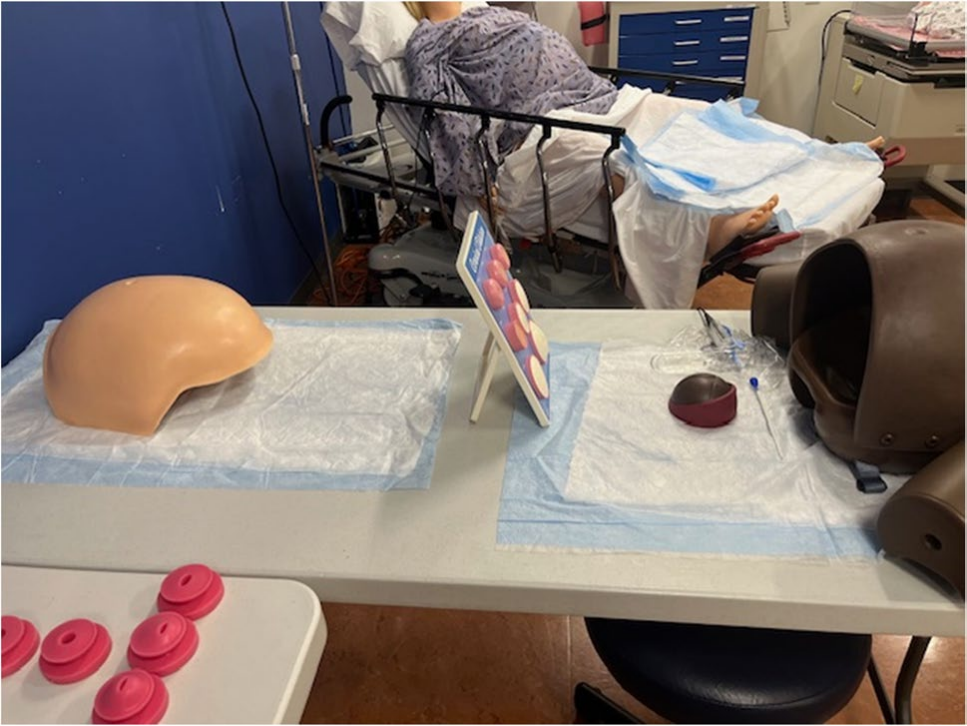
PROMPT flex birthing simulator assessed cervical dilation, effacement, fetal station, and fetal positioning. Vaginal swab indicated PROM. Obstetric abdominal palpation model evaluated Leopold maneuvers.

**Fig. 3:**
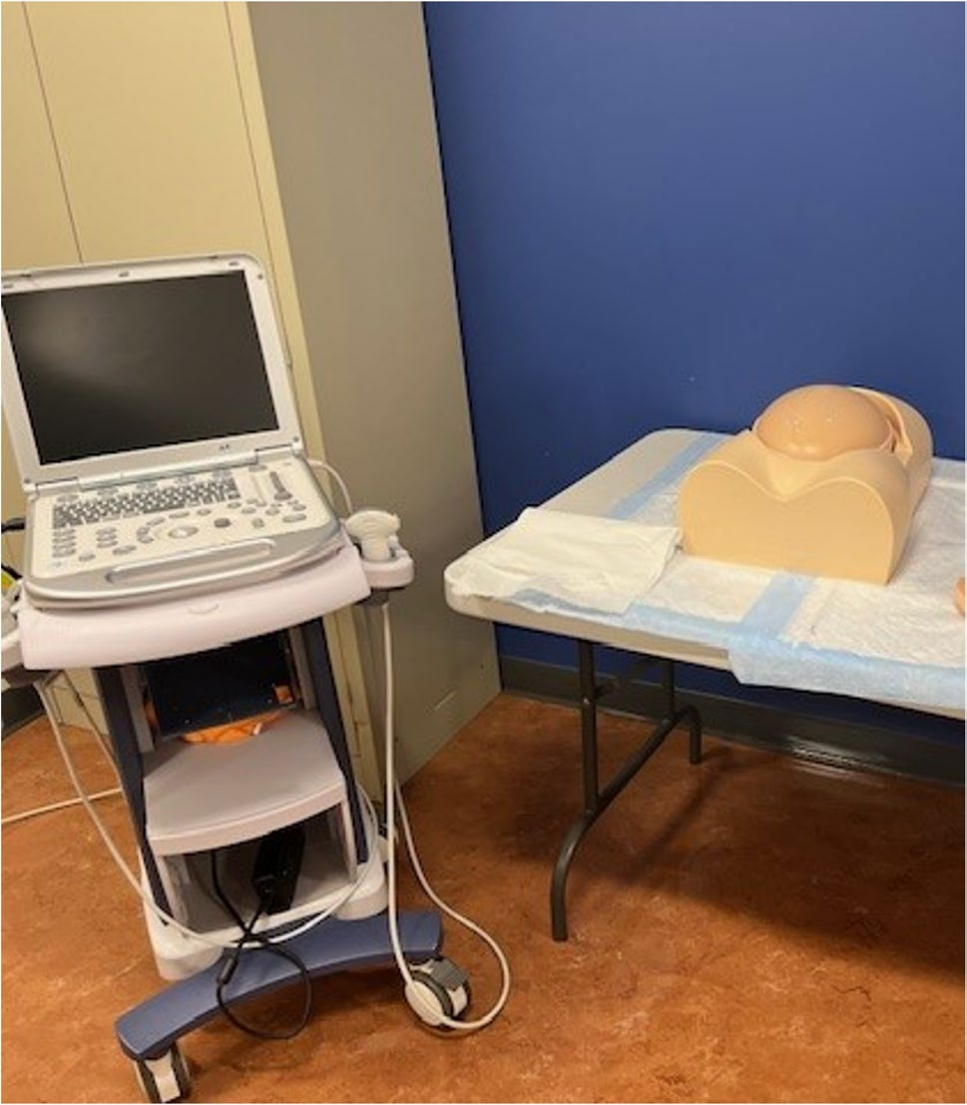
Fetus ultrasound examination phantom “SPACE FAN-ST” confirmed placental location and fetal positioning

**Fig. 4:**
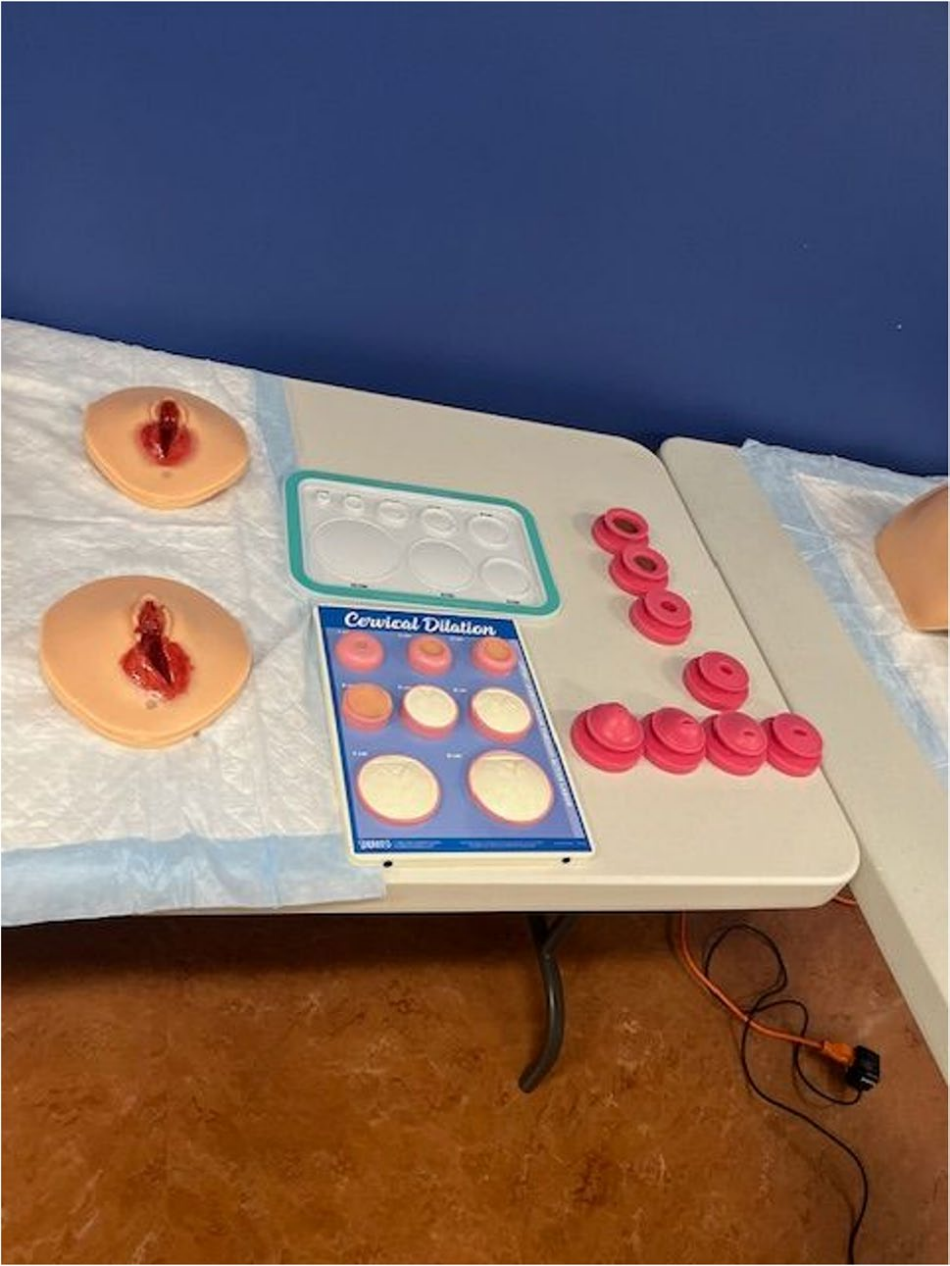
Laceration attachment from the VICTORIA S2200 and cervical dilation and effacement progression models

A high-fidelity childbirth simulation manikin **(**Fig. 1**)**was utilized during the obstetrical intrapartum simulated vaginal delivery experience to medically manage a patient who presents with premature rupture of membranes (PROM) and decreased fetal movement. The simulation case was designed to reinforce obstetrical knowledge and to allow students to practice a broad range of obstetrical history and physical examination skills along with clinical reasoning opportunities. The students conducted a directed medical history on the manikin. The manikin provided pertinent information that included a generally uncomplicated full-term pregnancy with several common clinical findings including Group B Streptococcus (GBS), PROM, and decreased fetal movement. According to the American College of Obstetricians and Gynecologists, approximately one in four women are colonized with GBS[6], and PROM occurs in roughly 8% of term pregnancies[7], making both important and frequently encountered conditions. The students performed a physical examination that included vital signs revealing an elevated blood pressure along with a pelvic exam that assessed cervical dilation and effacement along with fetal station and position. Next, they sampled replicated vaginal fluid to measure its pH and performed a fern test **(**Fig. 2**)**. Students then used a simulation model to perform Leopold maneuvers **(**Fig. 2**)** and conduct a transabdominal ultrasound confirming placental and fetal position **(**Fig. 3**)**. Students interpreted electronic fetal heart tracings throughout the encounter noting baseline heart rates, variability, both accelerations and decelerations and their associated complications. Students collaborated in a small group to formulate an initial assessment and management plan. Lab results were given for students to interpret which included a complete blood count, comprehensive metabolic panel, urinalysis, and a blood type and screen. “What, So What, Now What” debriefing method of learners by the simulation faculty facilitator reinforced obstetrical material and was conducted throughout the simulation experience. This method focuses on reflection of what is happening, why it matters, and what actions can be taken based on the experience. Lastly, a live vaginal delivery was performed on the high-fidelity childbirth simulation manikin along with delivery and inspection of the placenta. Replica models were used to grade postpartum lacerations **(**Fig. 4**)**. Apgar scores were performed on a life-like newborn manikin.

Students engaged in this exercise at the end of their didactic coursework including completion of the obstetrics and gynecology block in the clinical medicine course. Students completed a pre-survey (Supplementary File 1) and post-survey (Supplementary File 2). The pre- and post-simulation surveys were self-developed by the authors and informed by relevant themes in the literature on clinical preparation and confidence-building through simulation-based learning [8]. 

Pre-surveys were distributed three days prior to the activity, and post-surveys were collected immediately after the experience and remained open for two days. The pre-survey included five items, and the post-survey included four items plus one required short-answer response. With the exception of the qualitative item, all questions used a 5-point Likert scale (“Strongly Disagree” to “Strongly Agree”). Conceptually matched items were compared descriptively between pre- and post-surveys; however, formal statistical testing (e.g., Mann-Whitney U) was not performed due to differences in item wording and focus. Response frequencies and percentages were calculated, with “Agree/Strongly Agree” and “Disagree/Strongly Disagree” combined for analysis. A conventional qualitative content analysis was applied to 45 students’ optional narrative comments from the post-simulation survey, representing a subset of the 90 total survey respondents. This analytical approach was selected to allow categories and themes to emerge directly from participants’ words rather than from predefined theoretical perspectives [9]. Data were reviewed repeatedly for immersion, and meaning units were inductively coded to capture participants’ experiences and perceptions. Since some comments contained multiple ideas, coding was conducted at the level of individual statements to ensure each distinct concept was represented. Codes were then organized into categories and overarching themes through iterative comparison, allowing patterns to develop organically from the data. To contextualize the qualitative findings, the frequency and percentage of respondents who mentioned each theme were calculated (*n* = 90). Representative quotations were selected to illustrate key themes. All analyses were conducted using Microsoft Excel 2019. The study was deemed exempt by the Division of Research Institutional Review Board at the Philadelphia College of Osteopathic Medicine (approval #H24-061X, 09/04/2024).

## Results

A total of 90 students (59 from the Philadelphia campus and 31 from the Georgia campus) in the Class of 2025 participated in the obstetric labor and delivery simulation activity. Response rates were 98% for the pre-survey and 74% for the post-survey. The pre-survey evaluated students’ foundational knowledge of labor and delivery, relevant skill sets, and prior obstetrical simulation-based learning experiences. As shown in Table 1, 74.7% of participating students reported no prior experience with obstetrical simulation-based learning, and 85.7% reported never having used obstetrical simulation manikins. Furthermore, 93.4% of students reported that the simulation-based learning activity helped reinforce their clinical knowledge. Additionally, 92.3% stated it improved their hands-on clinical skills related to the labor and delivery process, and 93.4% indicated it enhanced their understanding and ability to apply these skills in real-life situations. It is also noteworthy that fewer than 12.1% of respondents selected “neither agree nor disagree” for all pre-survey questions.

**Table 1:** Pre-obstetrical student survey results. Response rate for labor & delivery pre-survey: 98% (88 out of 90 total students)

Pre-Survey Questions	Percentage of Students Who “Strongly Agree” or “Agree”	Percentage of Students Who “Neither Agree” nor “Disagree”	Percentage of Students Who “Strongly Disagree” or “Disagree”
I am familiar with obstetrical and gynecologic simulation-based learning experiences.	13.2	12.1	74.7
I have previous experience with the use of obstetrical and gynecologic simulation manikins.	6.6	7.7	85.7
I feel the obstetrical and gynecologic simulation-based learning experience will help reinforce my clinical knowledge.	93.4	6.6	0.0
I feel obstetrical and gynecologic simulation-based learning experience will help me with hands-on clinical skills.	92.3	7.7	0.0
I feel this obstetrical and gynecologic simulation experience will allow a better understanding and be able to apply my skills to a real-life situation.	93.4	6.6	0.0

In the post simulation results, 90.0% of participating students anticipate the experience will be useful in applying their knowledge to real patients, while 10% neither agree nor disagree **(**Table 2**)**. Of the 67 students that completed the post-survey, 94.3% reported the experience was appropriately designed for their knowledge and skill level, and 88.6% reported feeling comfortable with the material presented throughout the activity. Notably, 10.0% of students selected neither agree nor disagree with the statement, “The OB Simulation learning experience with the use of the manikin will be useful in applying my skills to real patients.” However, 92.8% of students reported that the simulation experience gave them increased confidence to work in a labor and delivery setting.

**Table 2:** Post-survey obstetrical simulation results. Response rate for labor & delivery post-survey: 74% (67 out of 90 total students)

Post-Survey Questions	Percentage of Students Who “Strongly Agree” or “Agree”	Percentage of Students Who “Neither Agree” nor “Disagree”	Percentage of Students Who “Strongly Disagree” or “Disagree”
The OB Simulation learning experience with the use of the manikin will be useful in applying my skills to real patients.	90.0	10.0	0.0
The OB simulation was designed for my specific level of knowledge and skill set.	94.3	5.7	0.0
I felt comfortable with the presented material throughout the simulation experience.	88.6	10.0	1.4
I felt the OB experience allowed me to enhance my confidence in working in a labor and delivery setting.	92.8	4.3	2.9

For conceptually matched items, agreement remained high pre- to post-simulation: reinforcing clinical knowledge yielded 93.4% in the pre-survey and 94.3% in the post-survey, hands-on/practical skills yielded 92.3% in the pre-survey and 90.0% in the post-survey, and understanding/applying skills yielded 93.4% in the pre-survey and 92.8% in the post-survey. Table 3 summarizes the pre- and post-survey responses for conceptually matched items, including the full Likert distribution.

**Table 3:** Pre- and post-survey responses for conceptually matched including the full likert distribution of agreement, neutrality, and disagreement

Concept	Pre-Survey Agree/ Strongly Agree (%)	Pre-Survey Neutral (%)	Pre-Survey Disagree/ Strongly Disagree (%)	Post-Survey Strongly Agree/ Agree (%)	Post-Survey Neutral (%)	Post-Survey Disagree/ Strongly Disagree (%)
Reinforce clinical knowledge	93.4	6.6	0.0	94.3	5.7	0.0
Hands-on/practical skills	92.3	7.7	0.0	90.0	10.0	0.0
Understanding & applying skills	93.4	6.6	0.0	92.8	4.3	2.9

Of the 90 students who completed the post-simulation survey, 45 students provided optional qualitative comments. Student feedback revealed five key themes, as shown in Table 4. The most prominent theme, *Preparation for Clinical Rotations*, was identified by 15.5% of students, who described the simulation as a positive experience that enhanced confidence and readiness for the clinical phase. The second most prominent theme, *Enhanced Knowledge Beyond the Classroom*, was identified by 12.2% of responding students with comments aligning to appreciation for applying theoretical concepts using obstetrical models. *Scheduling and Timing of Activity* was identified by 3.3% of the responding students indicating an appreciation for alignment of simulations more closely with coursework to optimize learning. Additionally, smaller but meaningful themes included *Facilitator Support* where 4.4% of students valued instructor guidance, and *Teamwork and Collaboration* where 3.3% of participants highlighted improved communication and team-based problem solving. Together, these findings underscore the simulation’s role in bridging theory and practice, enhancing clinical preparedness, and informing future curricular design.

**Table 4:** Qualitative response themes from the post-obstetrical survey. Total respondents *n* = 90

Themes	Number of Comments	% of Total Respondents	Description	Illustrative Quote
Enhanced Knowledge Beyond the Classroom	11	12.2	Students valued the simulation for reinforcing theoretical concepts and applying knowledge in a practical setting using simulation obstetrical models	“This simulation activity was great experience using knowledge learned in the classroom and trying to apply it to a ‘real life’ scenario.”
Preparation for Clinical Rotations	14	15.5	The simulation effectively enhanced students’ confidence and preparedness for clinical rotations. Students expressed overall positive experience with the simulation	“Overall, I thought this experience was very beneficial and will be helpful moving into clinical year.”
Scheduling and Timing of Activity	3	3.3	Students suggested that timing and scheduling of the activity should be altered to be closer to the didactic obstetric block	“I think the sim is a great addition to this block, the only thing I could suggest is moving the sim up a few weeks so it is closer to when the obstetrics block was happening!”
Facilitator Support	4	4.4	Students valued facilitator guidance and instructional support throughout the simulation	“The professor-led format was exactly what was needed for us to fully understand the simulation and get the most out of it”
Teamwork & Collaboration	3	3.3	Students noted improved collaboration and communications skills during the simulation	“I really enjoyed this opportunity to work as a team to talk through and use hands on skills for this before starting rotations.”

## Discussion

These findings highlight the strong impact of simulation-based learning and provide a foundation for the discussion of its broader implications. According to ARC-PA accreditation standards, programs are encouraged to remain innovative in curriculum design, delivery, and evaluation methods.² Obstetrical simulation represents one such innovation, offering a valuable opportunity to supplement supervised clinical practice in women’s health, particularly in areas that are encouraged but not mandated, such as labor and delivery exposure.² Through simulation, PA students gain exposure to acute, high-intensity labor and delivery scenarios in a controlled, safe environment, allowing them to review didactic obstetrical material, refine clinical skills, and demonstrate core clinical competencies.

Labor and delivery topics are consistently represented in standardized assessments such as the Women’s Health End of Rotation (EOR) exam, the Physician Assistant Clinical Knowledge Rating and Assessment Tool (PACKRAT), and the Physician Assistant National Certifying Examination (PANCE). Integrating obstetrical simulation into PA education provides an opportunity to reinforce didactic material through repetition, application, and hands-on skill development. This experiential approach not only supports kinesthetic learners but also promotes deeper engagement and knowledge retention compared to traditional, instructor-centered methods. By bridging theoretical knowledge with practical application, simulation-based learning has the potential to improve both clinical preparedness and examination performance. Future studies are warranted to evaluate the direct impact of obstetrical simulation on standardized assessment outcomes.

PA programs interested in incorporating obstetrical simulation into their curriculum can tailor simulation objectives to address specific programmatic needs. For instance, if institutional data reveals that students are scoring below benchmark in areas such as pregnancy complications, faculty can design simulation scenarios to target those deficiencies. Content can be customized to align with standardized exam topic lists and may include conditions such as breech presentation, shoulder dystocia, fetal distress, premature rupture of membranes, preterm labor, and umbilical cord prolapse. Additional complications directly affecting labor and delivery, such as gestational diabetes, incompetent cervix, placental abruption, placenta previa, and Rh incompatibility, may also be integrated to address gaps in both knowledge and clinical decision-making. Furthermore, the simulation can include the management of high-risk maternal conditions like preeclampsia, eclampsia, and pregnancy-induced hypertension during labor.

Expanding the scope of simulation to include perinatal, postnatal, and postpartum care may further enhance its educational value. Topics such as normal physiologic changes during the puerperium, perineal laceration repair, episiotomy care, postpartum hemorrhage management, and recognition and treatment of endometritis can be embedded into the simulation to support holistic learning. These additions would enable programs to bridge specific knowledge gaps while increasing student confidence in managing comprehensive obstetric care.

The relevance of this training is underscored by workforce trends. The number of physician assistants practicing in obstetrics and gynecology has increased in recent years, likely in response to a national decline in the number of OB/GYN physicians and midwives [10]. Given this growing demand, it is essential that PA graduates are confident and competent in obstetrical care. Encouragingly, the majority of survey respondents reported feeling confident in applying the skills acquired during the simulation to real-world clinical settings. Many also expressed that the experience enhanced their perceived ability to function effectively in a labor and delivery environment. Beyond technical skills, the simulation fostered interprofessional collaboration by encouraging team-based practice, a core competency in modern healthcare education.

Although the simulation activity was well-received, there remains room for improvement in curriculum integration. In this study, the simulation was delivered approximately two weeks prior to the end of the didactic phase, following instruction on obstetrics. While most students felt the timing was appropriate, 3.3% suggested they would have benefited more from participating in the simulation prior to obstetrical block testing. While this observation was not a prominent theme in the qualitative data, it underscores the importance of exploring the ideal timing for simulation. Future studies should assess whether simulation is most beneficial when introduced during the didactic phase, immediately following didactic instruction, or throughout clinical rotations.

### Limitations

This study has several limitations. Foremost, the innovative activity and data analysis was performed with a single institutional cohort from two regional campuses at the end of the didactic phase of the curriculum. Therefore, comparisons between other cohorts, institutions, and the clinical phases of the curriculum were not investigated. Another limitation of this pilot study is that identical questions were not utilized in the pre- and post-simulation surveys, which precluded direct comparison of specific responses across time points. Conceptually linked questions were consistent between surveys to explore perceptions and experiences. However, this design was intentionally selected to minimize recall and response bias that might occur if participants were presented with the same items at both time points. By tailoring the questions to the distinct pre- and post-simulation contexts, the study aimed to elicit more authentic reflections of participants’ baseline expectations and learning experiences. Future studies employing identical or parallel survey items could allow for a more rigorous quantitative assessment of change while maintaining strategies to limit bias.

Strengths of this study include a high pre-survey response rate of 98% and a high post-survey response rate of 74% among a relatively large cohort consisting of 90 participating students. This innovative obstetric activity is the first to be documented within PA education and can potentially bridge the gap of limited obstetrical clinical experiences.

## Conclusion

Benefits exist to incorporating obstetrical simulation in the PA curriculum. Given the rigors of obtaining women’s health clinical rotation sites that offer obstetric experiences, the implementation of obstetrical intrapartum simulated vaginal delivery provides a transformative approach to enhancing learning within the curriculum. This interactive activity offers the potential for PA students to develop their skills, enhance their preparedness, and build confidence in labor and delivery settings that may not be encountered during the clinical phase.

The success of this activity was confirmed by positive pre-survey and post-survey data. Healthcare educators may find benefit in the utilization of simulation based obstetric learning to help enhance clinical skills as well as promoting student engagement and teamwork in labor and delivery settings. Overall, obstetric simulation serves as a valuable supplement that reinforces knowledge and skills, creating new opportunities for learning within healthcare education.

## Supplementary Information

Supplementary Material 1: File 1- Pre-Obstetrical Simulation Survey. File 2- Post- Obstetrical Simulation Surgery.

## Data Availability

The datasets used and/or analyzed during the current study are available from the corresponding authors on reasonable request.

## Abbreviations

ARC-PA: Accreditation Review Commission on Education for Physician Assistant
EOR: End of Rotation
GBS: Group B Streptococcus
IRB: Institutional Review Board
OB/GYN: Obstetrics/gynecology
PA: Physician assistant
PACKRAT: Physician Assistant Clinical Knowledge Rating and Assessment Tool
PAEA: Physician Assistant Education Association
PANCE: Physician Assistant National Certifying Examination
PROM: Premature rupture of membranes
SBT: Simulation based training
