# Supplementary material for: Incorporating obstetrical simulation into physician assistant didactic curriculum
Source: BMC Med Educ. 2025 Dec 29;25:1731. doi: 10.1186/s12909-025-08342-w (PMC12750655; doi:10.1186/s12909-025-08342-w)
Supplement: Supplementary file 1 — Supplementary Material 1: File 1- Pre-Obstetrical Simulation Survey. File 2- Post- Obstetrical Simulation Surgery. [file 12909_2025_8342_MOESM1_ESM.docx]

**Supplementary File 1- Pre OB Simulation Survey**

**Question 1**- I am familiar with obstetrical and gynecologic simulation-based learning experiences.

Strongly Agree

Agree

Neither Agree nor Disagree

Disagree

Strongly Disagree

**Question 2**- I have previous experience with the use of obstetrical and gynecologic simulation manikins.

Strongly Agree

Agree

Neither Agree nor Disagree

Disagree

Strongly Disagree

**Question 3**- I feel the obstetrical and gynecologic simulation-based learning experience will help reinforce my clinical knowledge.

Strongly Agree

Agree

Neither Agree nor Disagree

Disagree

Strongly Disagree

**Question 4**- I feel obstetrical and gynecologic simulation-based learning experience will help me with hands-on clinical skills.

Strongly Agree

Agree

Neither Agree nor Disagree

Disagree

Strongly Disagree

**Question 5**- I feel this obstetrical and gynecologic simulation experience will allow a better understanding and be able to apply my skills to a real-life medical situation.

Strongly Agree

Agree

Neither Agree nor Disagree

Disagree

Strongly Disagree

**Supplementary File 2- Post OB Simulation Surgery**

**Question 1**- The OB simulation learning experience with use of the manikin will be useful in applying my skills to real patients.

Strongly Agree

Agree

Neither Agree nor Disagree

Disagree

Strongly Disagree

**Question 2-** The OB simulation was designed for my specific level of knowledge and skill set.

Strongly Agree

Agree

Neither Agree nor Disagree

Disagree

Strongly Disagree

**Question 3-** I felt comfortable with the presented material throughout the simulation experience.

Strongly Agree

Agree

Neither Agree nor Disagree

Disagree

Strongly Disagree

**Question 4-** I felt the OB experience allowed me to enhance my confidence in working in a labor and delivery setting.

Strongly Agree

Agree

Neither Agree nor Disagree

Disagree

Strongly Disagree

**Question 5 (Short Answer)-** Please enter any comments, positive or constructive, which you feel would help or enhance this experience. Please express these professionally.
